# Genetic and Epigenetic Regulation of *CCR5* Transcription

**DOI:** 10.3390/biology1030869

**Published:** 2012-12-13

**Authors:** Rutger J. Wierda, Peter J. van den Elsen

**Affiliations:** 1 Department of Immunohematology and Blood Transfusion, Leiden University Medical Center, Leiden, Albinusdreef 2, 2333 ZA, Leiden, The Netherlands; E-Mail: r.j.wierda@lumc.nl; 2 Department of Pathology, VU University Medical Center, De Boelelaan 1117, 1081 HV Amsterdam, The Netherlands

**Keywords:** transcription regulation, CCR5, epigenetics, DNA methylation, multivalent chromatin

## Abstract

The chemokine receptor CCR5 regulates trafficking of immune cells of the lymphoid and the myeloid lineage (such as monocytes, macrophages and immature dendritic cells) and microglia. Because of this, there is an increasing recognition of the important role of CCR5 in the pathology of (neuro-) inflammatory diseases such as atherosclerosis and multiple sclerosis. Expression of *CCR5* is under the control of a complexly organized promoter region upstream of the gene. The transcription factor cAMP-responsive element binding protein 1 (CREB-1) transactivates the *CCR5* P1 promoter. The cell-specific expression of *CCR5* however is realized by using various epigenetic marks providing a multivalent chromatin state particularly in monocytes. Here we discuss the transcriptional regulation of *CCR5* with a focus on the epigenetic peculiarities of *CCR5* transcription.

## 1. Introductions

CC chemokine receptor 5 (CCR5), a receptor for the CC chemokines macrophage inflammatory protein-1α (MIP-1α), macrophage inflammatory protein-1β (MIP-1β), and regulated and normal T cell expressed and secreted (RANTES), regulates trafficking of lymphoid cells such as memory/effector Th1 lymphocytes, or cells of the myeloid lineage (e.g., monocytes, macrophages, immature dendritic cells) and microglia [1,2,3]. As such, CCR5 is implicated in the pathogenesis of various inflammatory diseases such as atherosclerosis and multiple sclerosis [4,5,6,7]. This is illustrated in atherosclerotic mouse models, in which *Ccr5* knock-out mice show less neointima formation and an increase in production of the anti-inflammatory IL-10 molecule by vascular smooth muscle cells (vSMCs) [8,9]. One of the ligands for the CCR5 receptor, CCL5 or RANTES, has also been shown to be involved in unstable angina pectoris, which usually results from atherosclerosis [10]. Furthermore, CCR5 also functions as a co-receptor for HIV-1 [11,12,13]. Importantly, individuals with a mutated CCR5 receptor appear to be near-completely protected for HIV-1 infection [10,13]. Notably, CCR5 expression is markedly upregulated upon T cell activation, which allows the activated T cells to migrate towards site(s) of inflammation [14,15,16,17,18].

Upon encountering a pathogen, antigen-presenting cells will present the antigenic peptide to resting naive T cells, which results in the generation and activation of antigen-specific T cells [19,20]. After activation, the T cells migrate to the site of inflammation, guided by chemokine receptors [21]. Similarly, circulating monocytes are also attracted to inflammatory sites by chemokine receptors, where they then can differentiate into e.g., macrophages or microglia [22,23,24]. Atherosclerosis and multiple sclerosis are greatly characterized by inflammatory lesions, consisting of T cells and macrophages or microglia, respectively [25,26,27]. The chemokine receptor CCR5 is implicated in the pathogenesis of both of these diseases [8,9,28,29].

With CCR5 being implicated in various (inflammatory) diseases and as co-receptor for HIV-1 infection, much work has been put into the transcriptional regulation of *CCR5*. *CCR5* transcription is controlled by a complex promoter structure. Although a number of transcription factors, including CREB-1, have been shown to play a role in *CCR5* transcriptional regulation, its cell type specific expression is also controlled by epigenetic mechanisms. In some cell types the chromatin status of *CCR5* is rather peculiar in that it is hallmarked by various multivalent states. In this review we will discuss the regulation of *CCR5* expression, with the focus on epigenetic regulation and the possible pharmacological intervention in these epigenetic regulatory processes.

## 2. Genomic Organization

**Figure 1:**
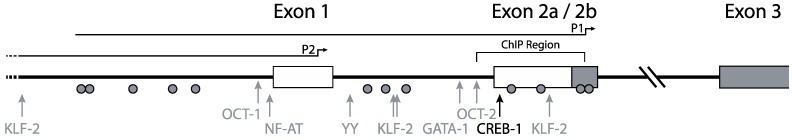
Promoter organization of the *CCR5* gene. *CCR5* transcription is initiated from two distinct promoters. Full-length transcripts arise from promoter P1 and truncated transcripts from promoter P2. The black arrow indicates the most responsive CREB-1 site. Other transcription factors attributed to *CCR5* transcription regulation are depicted with gray arrows. Grey circles indicate CpG residues. The region investigated for epigenetic regulation by chromatin immune precipitation is annotated as “ChIP region”. Adapted from [31].

Mummidi *et al.* previously elucidated the organization and promoter usage of the *CCR5* gene (Figure 1) [16,30]. Two distinct functional promoter regions for the *CCR5* gene were identified using luciferase-reporter constructs containing *CCR5* regulatory regions: a downstream promoter region, nowadays designated as P1, and an upstream promoter region, designated P2. The P2 promoter in front of exon 1 was identified as a weaker promoter than the P1 promoter, which is located near exon 2b (Figure 1) [16]. Using 5' RACE and RT-PCR the authors found four exons and two introns in the genomic organization of *CCR5*. The authors suggested that two full-length transcripts arose from promoter P1 and numerous truncated transcripts (*i.e.*, missing exon 1) arose from promoter P2. Between exon 2a and exon 2b there is no intron. Both the truncated and the full-length transcripts give rise to the full length CCR5 protein. The two different full-length transcripts designated CCR5A and CCR5B differ by 235 nucleotides, corresponding to a lack of exon 2 in the CCR5B transcript.

Both P1 and P2 lack the canonical TATA and CCAAT motifs, although in P1 a non-classical TATA-box can be found. Unlike other TATA-less promoters, the CCR5 promoters have a relatively low CpG content.

## 3. CCR5 Regulation by Transcription Factors

In their initial characterization of the *CCR5* promoter, Liu and coworkers suggested that *CCR5* transcription could be up-regulated by NF-κB [32]. Indeed, others and we found several potential binding sites for NF-κB in the *CCR5* P1-promoter [33]. However, the results of the study by Kuipers *et al.* indicate that *CCR5* expression is neither induced nor modulated by NF-κB [33]. In addition, these authors also found binding sites for interferon regulatory factors (IRFs) and CREB-1 in the *CCR5* P1- and P2-promoters. Like for NF-κB and in contrast to CREB-1, Kuipers *et al.* could not establish a role for the IFN-γ induced regulatory pathway in *CCR5* transcription. By using various reporter assays, as well as by competition for CREB-1 binding-sites by inducible cAMP early repressor (ICER), which is induced by forskolin treatment, the authors concluded that *CCR5* transcription is regulated by CREB-1 [33]. More recently, Banerjee *et al.* also showed that in the TF-1 human bone marrow progenitor cell line, *CCR5* is regulated at the transcriptional level by the cAMP/PKA/CREB pathway [34].

In line with an important role for CREB-1 in *CCR5* expression are the transcription levels of CREB-1 isoforms and ICER in activated versus naïve CD4^+^ T cells. Upon activation of CD4^+^ T cells, the relative transcription levels of CREB-1 isoforms increase whereas transcription levels of ICER decrease [31].

The site most responsive to the CREB-1-mediated transactivation is located in the downstream P1-promoter (Figure 1). Promoter constructs using the upstream promoter however were unresponsive to CREB-1. This suggests a repressive function of the upstream promoter region. This hypothesis is underscored by the fact that the longest downstream promoter construct, including part of the upstream region, is less responsive to CREB-1 than truncated downstream promoter constructs. These findings corroborate those of others that mapped a repressive element, corresponding to a region upstream, affecting the *CCR5* promoter [32,35,36]. Besides an important role for CREB-1 in the transcriptional regulation of *CCR5*, several other studies have revealed also a contribution for Oct1 and Oct2 [15,37], NF-AT [32], YY1 [38], GATA-1 [39], and KLF2 [40] in the modulation of *CCR5* transcription.

## 4. Epigenetic Regulation

In addition to the transcription factors discussed above, most if not all genes are also regulated at the transcriptional level by epigenetic processes. Since *CCR5* is only expressed in a subset of T lymphocytes, monocytes, macrophages, or dendritic cells the contribution of epigenetic mechanisms in the cell-type specific transcriptional regulation of *CCR5* was proposed and investigated [31,33].

In the cell nucleus, DNA is present as a protein-DNA complex called chromatin. The basic repeating unit of chromatin is the nucleosome, which consists of 146bp of DNA wrapped around an octamer of histones, comprising pairs of the core-histones H2A, H2B, H3 and H4. The structure of the chromatin can be altered by epigenetic regulation through modification of DNA and of histones. In this way, epigenetic regulation controls gene transcription by modifying the chromatin architecture in such a way that it influences the accessibility of DNA for transcription factors. Note, these epigenetic modifications are reversible allowing the chromatin structure to switch between open and closed states. This form of transcriptional control is thought to form also the basis for cell-to-cell inheritance of gene expression profiles [41]. Epigenetic modifications include methylation of DNA at CpG residues in gene promoters and post-translational modifications of histone tails such as acetylation and methylation [42].

DNA methylation is perhaps one of the best-studied epigenetic modifications. CpG residues are underrepresented in the human genome but are highly enriched in so-called CpG islands in most gene promoters [43,44]. As a general rule of thumb, gene expression is associated with unmethylated CpGs in gene promoters, while CpG methylation in gene promoters is associated with transcriptional repression [45]. More recently, the involvement of additional CpG containing regions, the so-called CpG island shores, at more distal locations from gene promoters in gene transcription has become apparent [46]. Mechanisms that underlie gene repression by histone methylation involve, amongst others, tri-methylation of histone H3 at lysine 9 (3MeK9H3) and at lysine 27 (3MeK27H3), and of histone H4 at lysine 20 (3MeK20H4) in proximal promoter chromatin [47,48,49,50]. Counteracting these repressive modifications are the transcriptionally permissive modifications tri-methylation of histone H3 at lysine 4 (3MeK4H3) and histone acetylation [50,51,52].

Together these modifications form a “histone code”, like the genetic code, that controls transcription levels of genes [53]. As mentioned above, one important trait of epigenetic regulation is that modifications to DNA and to histone tails are reversible. Furthermore, these activities are functionally linked [54].

### 4.1. “Classical” Epigenetic Regulation

In the case of the *CCR5* locus, non-expressing cells such as Jurkat (a T leukemia cell line) cells have a densely methylated promoter. Conversely, cells on which CCR5 is highly expressed display a virtually unmethylated CCR5 promoter region [31].

**Figure 2:**
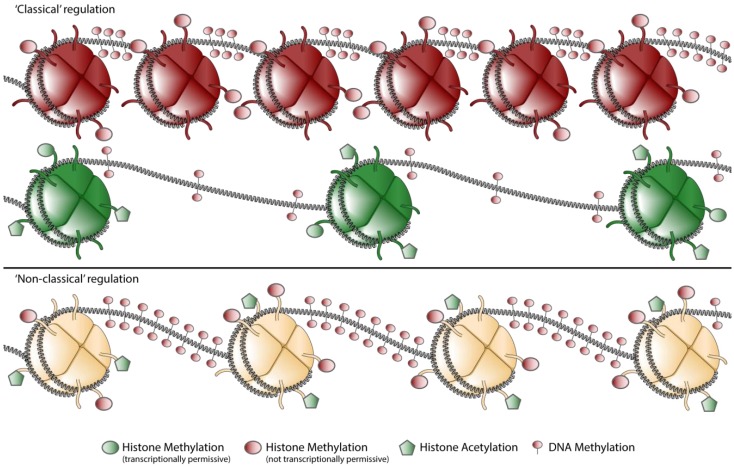
Schematic representation of chromatin states encountered in the *CCR5* locus. Chromatin can be marked by mainly repressive or mainly permissive marks, regarded as the classical euchromatin (green) and heterochromatin (red) states (“classical” regulation). Nowadays it is widely appreciated that more complex forms of chromatin exist, hallmarked by both repressive and permissive marks in the same locus (“non classical” regulation). Note: For clarity DNA-methylation is drawn on the internucleosomal-DNA, whereas it has been shown that methylated DNA co-localizes also with nucleosomes [59].

In a traditional view of epigenetic regulation, chromatin could either be in open (euchromatin) or closed (heterochromatin) conformation (Figure 2) [55,56]. Intermediate chromatin states were thought to be a transition between either hetero- or euchromatin and were thought to be only short lived [57]. When transcription was occurring, a gene promoter was enriched in acetylated lysine 9 in histone H3 (AcK9H3) and 3MeK4H3, whereas transcriptionally silenced genes where enriched in 3MeH3K27 and 3MeH3K9 [52,58].

When comparing activated and naïve CD4^+^ T cells, the *CCR5* locus displays such a classical pattern [31,33]. Naïve CD4^+^ T cells are characterized be relatively low levels of AcH3 and relatively high levels of the repressive mark 3MeK20H4. When compared to activated T cells, naïve T cells also contain high levels of 3MeK27H3. Upon activation the repressive marks in the chromatin of the CCR5 promoter are replaced by activating marks. Chromatin of the CCR5 promoter in activated CD4^+^ T cells is almost exclusively covered with the activating marks AcH3 and 3MeK4H3. In other cell types however, the chromatin makeup of the *CCR5* locus is more complex.

### 4.2. “Non-classical” Epigenetic Regulation

Bivalent chromatin structures, composed of both 3MeK4H3 and 3MeK27H3, were first found in embryonic stem cells. In these cells, bivalent states marked genes encoding transcription factors that play an essential role in development. It was proposed that these bivalent states would silence developmental genes while keeping them poised for activation. Upon differentiation, this bivalent state would be lost [60]. Later work not only identified bivalent, but also tri- and tetravalent chromatin states [61]. Furthermore, multivalent chromatin states where found to also occur in differentiated cells [31,61]. Lastly it turned out that bivalent, “poised” chromatin, is not completely silenced as proposed initially. Some transcripts arise from multivalent chromatin, thus multivalent chromatin might be a mechanism for transcriptional fine-tuning [62]. It is thought therefore that these multivalent states may be of importance for the control of gene expression in the activation of T cells and the differentiation of monocytes [62]. Chromatin Immunoprecipitation (ChIP) experiments performed on the *CCR5* promoter region revealed that this promoter is extensively covered by multivalent marks [31].

In CCR5-expressing monocytes, the *CCR5* promoter is covered with high levels of AcH3, but also with relative high levels of 3MeK9H3 and 3MeK27H3 and an intermediate level of 3MeK20H4. The histone modifications in monocytes are accompanied with relatively high levels of DNA methylation (Figure 2). Yet despite all these repressive modifications monocytes show *CCR5* transcription levels that are markedly higher then naïve CD4^+^ T cells, albeit at a lower level than in activated CD4^+^ T cells [31].

Monocytes have the ability to differentiate into macrophages and dendritic cells. Upon differentiation CCR5 expression is lost. The fact that monocytes display high amounts of repressive marks in conjunction with histone acetylation may reflect the potential to rapidly shut down *CCR5* transcription upon differentiation. The observed multivalent chromatin state of *CCR5* as such might reflect the central role of CCR5 in the regulation of lymphoid cell trafficking.

### 4.3. Epigenetic Intervention

Epigenetic modifications are reversible and can be modulated by small molecule inhibitors. With *CCR5* cell-type specific transcription being achieved by epigenetic regulation, small molecule inhibitors could modulate *CCR5* transcription. Jurkat cells do not express CCR5, however it was demonstrated by using promoter constructs that Jurkat cells contain all the necessary transcription factors to achieve *CCR5* transcription [32].

**Figure 3:**
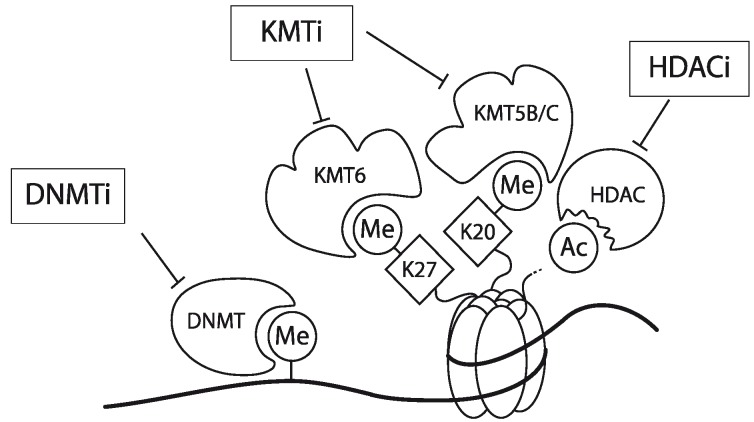
Schematic representation of the working mechanism for the pharmacological intervention in *CCR5* transcription. Re-expression was achieved by combinatorial treatment with a DNMT inhibitor (DNMTi, Zebularine), a lysine methyltransferase inhibitor (KMTi, DZNep) and a HDAC inhibitor (HDACi, MS-275). Adapted from [31].

In Jurkat cells, DNA of the *CCR5* promoter is densely methylated. Furthermore, chromatin encompassing the promoter region hardly contains AcH3 and is devoid of 3MeK4H3. Finally, the *CCR5* promoter is enriched for the repressive chromatin marks 3MeK9H3, 3MeK27H3 and 3MeK20H4. Using a combination of a DNMT inhibitor (Zebularine), a lysine methyltransferase inhibitor (DZNep) and a histone deacetylase (HDAC) inhibitor (MS-275; specific for the class 1 HDACs 1 and 3 [63,64,65]) the expression of the *CCR5* gene could be activated in Jurkat cells but also in other CCR5-deficient leukemic T cell-lines (Figure 3). Interestingly mono-treatment with these inhibitors showed only a very small effect [31]. Matalon *et al.* also have shown that treatment of CD4^+^ T cells and monocytes with HDAC inhibitors modulates the expression of CCR5 in these cell types [66]. Together, these observations underscore that *CCR5* transcription is actively controlled by epigenetic mechanisms. It should be noted however, that these inhibitors act genome-wide and as such targeting these inhibitors to specific genes may prove to be challenging. Meanwile, some of these epigenetic inhibitors have been in clinical use already for decades (e.g., for the management of epilepsy with Depakene, or valproic acid), despite the fact that they act genome-wide and they seem to be well tolerated [67,68].

## 5. Conclusions

Transcription of *CCR5* is achieved by a complex interplay of transcription factors and various forms of epigenetic regulation. Cell type-specific transcription of *CCR5* is achieved by epigenetic regulation. Epigenetic regulation also allows for rapid transcriptional shutdown or initiation of the *CCR5* locus upon differentiation of various cell types. This epigenetic regulation is a perfect entry point for pharmaceutical intervention. Given the importance of CCR5 in numerous inflammatory diseases and as co-receptor for HIV-1 gaining entrance into cells, pharmaceutical intervention in the epigenetic regulation of *CCR5* transcription may prove to be valuable in future disease management.
